# Loss of heterozygosity at chromosome 1p in different solid human tumours: association with survival

**DOI:** 10.1038/sj.bjc.6690234

**Published:** 1999-03

**Authors:** G Ragnarsson, G Eiriksdottir, J Th Johannsdottir, J G Jonasson, V Egilsson, S Ingvarsson

**Affiliations:** Department of Pathology, University and National Hospital of Iceland, P.O. Box 1465, Reykjavik, IS-121, Iceland

**Keywords:** cancer, chromosome 1p, loss of heterozygosity, survival statistics, tumour-suppressor gene

## Abstract

The distal half of chromosome 1p was analysed with 15 polymorphic microsatellite markers in 683 human solid tumours at different locations. Loss of heterozygosity (LOH) was observed at least at one site in 369 cases or 54% of the tumours. LOHs detected ranged from 30–64%, depending on tumour location. The major results regarding LOH at different tumour locations were as follows: stomach, 20/38 (53%); colon and rectum, 60/109 (55%); lung, 38/63 (60%); breast, 145/238 (61%); endometrium, 18/25 (72%); ovary, 17/31 (55%); testis, 11/30 (37%); kidney, 22/73 (30%); thyroid, 4/14 (29%); and sarcomas, 9/14 (64%). High percentages of LOH were seen in the 1p36.3, 1p36.1, 1p35–p34.3, 1p32 and 1p31 regions, suggesting the presence of tumour-suppressor genes. All these regions on chromosome 1p show high LOH in more than one tumour type. However, distinct patterns of LOH were detected at different tumour locations. There was a significant separation of survival curves, with and without LOH at chromosome 1p, in the breast cancer patients. Multivariate analysis showed that LOH at 1p in breast tumours is a better indicator for prognosis than the other variables tested in our model, including nodal metastasis. © 1999 Cancer Research Campaign


*British Journal of Cancer* (1999) **79**(9/10), 1468–1474
© 1999 Cancer Research Campaign
Article no. bjoc.1998.0234

### Loss of heterozygosity at chromosome 1p in different solid human tumours: association with survival

**G Ragnarsson, G Eiriksdottir, JTh Johannsdottir, JG Jonasson, V Egilsson and S Ingvarsson**

Department of Pathology, University and National Hospital of Iceland, P.O. Box 1465, IS-121 Reykjavik, Iceland

**Summary** The distal half of chromosome 1p was analysed with 15 polymorphic microsatellite markers in 683 human solid tumours at different locations. Loss of heterozygosity (LOH) was observed at least at one site in 369 cases or 54% of the tumours. LOHs detected ranged from 30–64%, depending on tumour location. The major results regarding LOH at different tumour locations were as follows: stomach, 20/38 (53%); colon and rectum, 60/109 (55%); lung, 38/63 (60%); breast, 145/238 (61%); endometrium, 18/25 (72%); ovary, 17/31 (55%); testis, 11/30 (37%); kidney, 22/73 (30%); thyroid, 4/14 (29%); and sarcomas, 9/14 (64%). High percentages of LOH were seen in the 1p36.3, 1p36.1, 1p35–p34.3, 1p32 and 1p31 regions, suggesting the presence of tumour-suppressor genes. All these regions on chromosome 1p show high LOH in more than one tumour type. However, distinct patterns of LOH were detected at different tumour locations. There was a significant separation of survival curves, with and without LOH at chromosome 1p, in the breast cancer patients. Multivariate analysis showed that LOH at 1p in breast tumours is a better indicator for prognosis than the other variables tested in our model, including nodal metastasis.

**Keywords:** cancer; chromosome 1p; loss of heterozygosity; survival statistics; tumour-suppressor gene

The majority of invasive human solid tumours are considered to be sporadic. Accumulation of multiple genetic alterations play a major part in tumorigenesis of these tumours. Mutations cause some genes (proto-oncogenes) to gain function while other genes [tumour-suppressor genes (TSG)] sustain loss of function (for review see Haber and Harlow, 1997). The localization of these genes to specific chromosome regions is currently a major area of study in cancer research. Loss or inactivation of TSG has been shown to be a major feature in the genesis of the solid cancers examined so far (Cavenee and White, 1995). As a full inactivation of a gene usually requires the silencing of both its alleles, the first inactivating mutation is, by inference, recessive. This first mutation can be somatic or passed through the germ line. The second mutation is somatic and proceeds through a chromosomal mechanism, which leads to loss of the wild-type allele (loss of heterozygosity) or replacement of the wild type allele by the mutant allele. The result is a complete absence of the normal protein product.

Loss of heterozygosity (LOH) on the short arm of chromosome 1 in solid human tumours has been reported in the following cancer types: breast cancer, neuroblastoma, mesothelioma, melanoma, testis cancer, liver cancer, stomach cancer, phaeochromocytoma, thyroid cancer, meningioma, colorectal cancer, endometrial cancer and Wilms tumour (Mathew et al, 1987; Dracopoli et al, 1989; Chen et al, 1992; Bardi et al, 1993; Taguchi et al, 1993; Bello et al, 1994; Bieche et al, 1994; Mathew et al, 1994; Stock et al, 1994; Yeh et al, 1994; Caron et al, 1995; Kuroki et al, 1995; Munn et al 1995; White et al, 1995; Di Vinci et al, 1996; Ezaki et al, 1996; Ragnarsson et al, 1996; Ogunbiyi et al, 1997; Vargas et al, 1997; Arlt et al, 1996; Steenman et al, 1997). All of these studies strongly indicate that TSGs may be located on the short arm of chromosome 1. A number of studies have shown a significant association between LOH at 1p and prognostic factors. The distal half of chromosome 1p from 1p31.1 to 1p36.3 is among the regions on 1p that have shown frequent LOH. At least two studies have shown an association between LOH at 1p and amplification of the N-*myc* proto-oncogene in breast tumours and neuroblastoma (Bieche et al, 1994; Caron et al, 1995). There seems to be an association between LOH at 1p and poor prognosis in patients with neuroblastoma (Caron et al, 1996), breast cancer (Ragnarsson et al, 1996) and colon cancer (Ogunbiyi et al, 1997). LOH at 1p was shown to be an early event in the carcinogenesis of breast cancer (Munn et al, 1995), liver cancer (Kuroki et al, 1995) and colorectal cancer (Di Vinci et al, 1996). One study indicates that LOH at 1p is a late event in melanomas (Dracopoli et al, 1989). Introduction of chromosome 1p36 into colon cancer cell line suppresses tumorigenic behaviour (Tanaka et al, 1993).

In this study we made an attempt to discover whether LOH patterns were similar in different types of human solid cancers. We screened 682 human solid tumours from 20 different locations for loss of heterozygosity at chromosome 1p, using 15 highly polymorphic microsatellite markers focusing on 1p31-pter. Furthermore, we tested whether there was an association between LOH at 1p and overall patient survival and clinico-pathological variables.

### MATERIALS AND METHODS

### Patients and tumour material

Fresh tumour samples from 683 primary solid tumours and normal tissue samples from the same patients were obtained on the day of surgery, immediately frozen and stored at – 70°C. The tumours were from the following locations (number of tumours investigated in parentheses): breast (238); colon and rectum (109); kidney (73); lung (63); stomach (38); ovary (31); testis (30); endometrium (25); thyroid (14); sarcoma (14); lymphoma (8);

*Received 19 May 1998*
*Revised 1 October 1998*
*Accepted 14 October 1998*

*Correspondence to:* S Ingvarsson

**Table 1** Information about the markers used in this study

| Marker | Distance from D1S243 (cM)[a] | Location | Informative samples | Tumours with LOH | % LOH |
|---|---|---|---|---|---|
| D1S243 | – | 1p36.3 | 376 | 70 | 20 |
| D1S468 | 6.4 | 1p36.3 | 340 | 86 | 25 |
| D1S214 | 16.8 | 1p36.3 | 396 | 84 | 21 |
| D1S228 | 33.2 | 1p36.1 | 333 | 65 | 20 |
| D1S507 | 39.1 | 1p36.1 | 387 | 74 | 19 |
| D1S436 | 43.6 | 1p36.1 | 258 | 59 | 23 |
| D1S233 | 65.6 | 1p35 | 191 | 43 | 23 |
| D1S201 | 66.7 | 1p35 | 246 | 53 | 22 |
| D1S496 | 69.4 | 1p34.3 | 143 | 44 | 31 |
| D1S209 | 98.8 | 1p32–p33 | 471 | 74 | 16 |
| D1S216 | 110.1 | 1p32–p33 | 342 | 58 | 17 |
| D1S207 | 120.5 | 1p32–p33 | 513 | 103 | 20 |
| D1S488 | 121.0 | 1p32–p33 | 420 | 116 | 28 |
| D1S167 | 128.4 | 1p31 | 426 | 104 | 24 |
| D1S435 | 131.8 | 1p31 | 465 | 127 | 27 |

[a]The markers have been mapped by Genethon from D1S243.

**Table 2** Multivariate analysis [proportional-hazard (Cox) regression] of survival in **A** 238 breast cancer patients and **B** 109 colorectal cancer patients

| Parameter | Univariate *P*-value | Multivariate *P*-value | RR[a] 95% CI |
|---|---|---|---|
| **A** | | | |
| LOH at 1p | < 0.001 | < 0.001 | 2.7 (1.5–4.9) |
| Axillary nodal involvement | 0.024 | 0.015 | 1.8 (1.1–3.0) |
| Tumour size[b] | < 0.001 | 0.381 | 1.3 (0.73–2.3) |
| S-phase fraction[c] | 0.020 | 0.071 | 1.6 (0.96–2.7) |
| Progesterone receptor[d] | 0.040 | 0.371 | 1.3 (0.76–2.1) |
| **B** | | | |
| LOH at 1p | 0.089 | 0.055 | 1.9 (0.99–3.6) |
| Dukes grade[e] | 0.024 | 0.017 | 2.2 (1.2–4.4) |
| Differentiation[f] | 0.348 | 0.287 | 1.5 (0.70–3.4) |

[a]RR = relative risk of dying in the multivariate analysis. [b]Tumour size > 2 cm. [c]S-phase fraction > 7%. [d]Progesterone receptor < 25 fmol $mg^{-1}$ protein. [e]Dukes grade C and D. [f]Differentiation grade III.

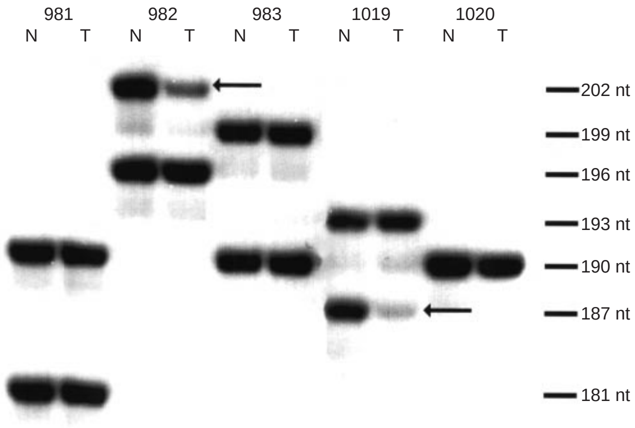

**Figure 1** The picture demonstrates a trinucleotide repeat polymorphism in five matched normal (N) and tumour (T) tissues from breast cancer patients. The microsatellite marker D1S488 was used in PCR analysis and the amplified products separated by electrophoresis in a 6.5% polyacrylamide 8 *M* urea denaturing gel. Case numbers are shown at the top. Numbers to the right indicate the size (in nucleotides) of the PCR product. Deletion can be seen in tumours 982 and 1019. The fact that there is not a complete loss of allele in these tumours is most probably due to contamination from normal DNA in the tumour sample. The tumours 981 and 983 have normal heterozygous allele patterns and patient sample 1020 is a homozygote oesophagus (6); liver (6); mouth (5); brain (5); pancreas (4); prostate (3); skin (3); adrenal gland (2); and unknown origin (6). In the case of the breast cancer patients, peripheral blood leukocytes were the source of normal DNA. Breast tumours were collected from the year 1987 to 1994 but other solid tumours from 1991 to 1997. All information about the tumours, e.g. size, type, age at diagnosis, histology and node status, was recorded at the Department of Pathology, University Hospital of Iceland.

### *DNA extraction and analysis*

Tumour and normal DNA was extracted from the tissue samples with proteinase K using a method developed for paraffin-embedded tissue (Smith et al, 1992). In the case of the breast samples, normal DNA was extracted from peripheral blood leukocytes by a salting-out method (Miller et al, 1988) and tumour DNA was extracted according to standard protocols. Paired blood and tumour DNA was subjected to PCR analysis. DynaZyme™ polymerase (from Finnzymes Oy, Espoo, Finland) was used in the buffer solution provided by the manufacturer. Samples were subjected to 35 cycles of amplification, consisting of 30 s at 94°C, 40 s at 55°C and 30 s at 72°C, followed by 10 min at 72°C. The markers used (Table 1) were obtained from Research Genetics (Huntsville, AL, USA). Primers were elongated by terminal-transferase in 40 mM K-HEPES/1 mM $CoCl_2$ buffer at pH 7.2 and 37°C over night. PCR products were separated on 6.5% polyacrylamide 8 M urea sequencing gels and transferred to a Hybond-$N^+$ nylon film (Amersham, Aylesbury, UK). They were then hybridized for at least 2 h at 42°C with the elongated primers, covalently labelled with peroxidase (ECL kit, Amersham, Aylesbury, UK). The membranes were washed once in 3 × SSC/0.1% SDS at 39–42°C and then twice in 0.2 × SSC at 39–42°C. After washing, the membranes were bathed in a detection reagent containing $H_2O_2$, luminol and an enhancer (ECL kit, Amersham, Aylesbury, UK) for 1 min at room temperature and signals were detected on DUPONT Cronex-4 film. Any absence or significant decrease (more than 50%) in the intensity of one allele relative to the other was considered LOH (Figure 1).

### *Statistical analysis*

A chi-squared test was used to assess the relationship between LOH at 1p and prognostic variables. In the breast tumours LOH at 1p was compared with node status, tumour size, histological type, age of diagnosis, steroid receptor content, S-phase fraction and tumour ploidy. In the other tumours LOH at 1p was compared with node status, tumour size and tumour differentiation. Survival curves were calculated according to the method of Kaplan and Meier (Kaplan and Meier, 1958). Tests of difference between curves were made with the log-rank test for censored survival data (Mantel, 1960). Multivariate analysis was performed with Cox's partially nonparametric regression model (Cox, 1972). The Survival Tools for Statview Package (Abacus Concepts, Inc., Berkeley, CA, USA) was used for the statistical analysis.

© Cancer Research Campaign 1999

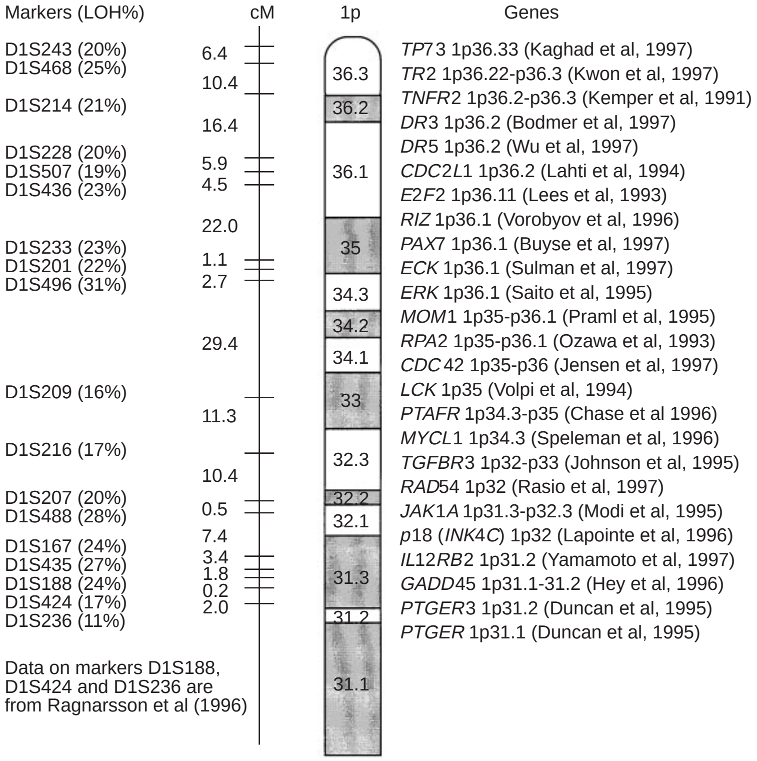

**Figure 2** Information about individual markers used in the study, their localization and percentage of LOH in tumour samples tested. The information for markers D1S188, D1S424, and D1S236 is from our earlier work with breast cases. There is also information about possible TSG and their location on chromosome 1p

### RESULTS

### Loss of heterozygosity at chromosome 1p

Fifteen markers were used for the analysis of microsatellite polymorphism at the distal half of chromosome 1p in 683 primary tumours. Representative results are shown in Figure 1. LOH with at least one marker was detected in 369 (54%) while 46 tumours showed LOH at all informative markers, suggesting complete deletion of this region on chromosome 1p (132 cM). Figure 2 shows LOH at different markers for all tumour samples tested, location of the markers and possible TSGs. There was a difference in LOH frequency between tumours of different locations. The types where LOH was detected in over 50% of the tumours were: endometrium, 18/25 (72%); sarcoma, 9/14 (64%); breast, 146/238 (61%); lung, 38/63 (60%); colon and rectum, 60/109 (55%); ovary, 17/31 (55%); and stomach, 20/38 (53%). The LOH frequency detected in thyroid, kidney and testis tumours was lower, i.e. 4/14 (29%), 22/73 (30%), and 11/30 (37%) respectively. There were less than 10 samples of each of the following tumour types, so the LOH frequency is of little reliability: mouth (2/5); oesophagus (2/6); liver (3/6); pancreas (2/4); prostate (2/3); melanoma (3/3); brain (2/5); phaeochromocytoma (1/1); adrenocortical cancer (1/1); lymphoma (5/8); and unknown origin (2/6).

Figure 3 shows the frequency of LOH detected in 10 different solid tumour locations, where the highest number of samples was available. The pattern of LOH is similar but several differences can be detected. Four peaks of LOH are detected with markers D1S468, D1S507, D1S488 and D1S435 in stomach tumours (Figure 3A). The two latter markers also show elevated LOH in tumours of other tissues, e.g. colorectum, lung and breast, as well as in sarcomas (Figure 3B, C, J, I). Furthermore, the D1S488 marker shows the highest detected LOH for an individual marker in this study in sarcomas at 63% (Figure 3I). Markers D1S243 and D1S436 also show high LOH in sarcoma, 50% and 60%, respectively (Figure 3I). Interestingly, the highest detected LOH in ovarian cancer is also by the D1S436 marker (Figure 3E). The D1S468 marker also has a high LOH in endometrial cancer, together with an elevation of LOH at markers D1S201 and D1S167 (Figure 3D). There is an interesting difference in the LOH pattern of markers D1S488, D1S167 and D1S435 in endometrial cancer compared with most of the other tumours. Low frequency was detected with all markers in testis, renal, and thyroid cancer, with the exception of an elevation of LOH detected by marker D1S216 in thyroid cancer (Figure 3F, G, H). The pattern of microdeletions in breast and colorectal tumours is consistent with the smallest region of overlap (SRO) in five regions: 1p36.3, 1p36.1, 1p35, 1p32 and 1p31 (Figure 4). A low number of microdeletions and the complex pattern of LOH in lung tumours makes mapping of SRO difficult. A complex pattern of LOH and loss of all markers analysed is more frequently detected in lung and colorectal tumours than tumours of breast and kidney. The fraction of complete loss of chromosome 1p31-pter (all informative markers with LOH) was as following: testis, 0/30 (0%); breast, 6/238 (2.5%); kidney, 3/73 (4.1%); stomach, 2/38 (5.3%); thyroid, 1/14 (7.1%); colon/rectum, 14/109 (13%); lung, 9/63 (14%); ovaries, 5/31 (16%); endometrium, 5/25 (20%); and sarcoma, 3/14 (21%). The mapping of the renal tumour samples indicates two SROs in the 1p36.3–p36.1 region, distinct from the

 © Cancer Research Campaign 1999

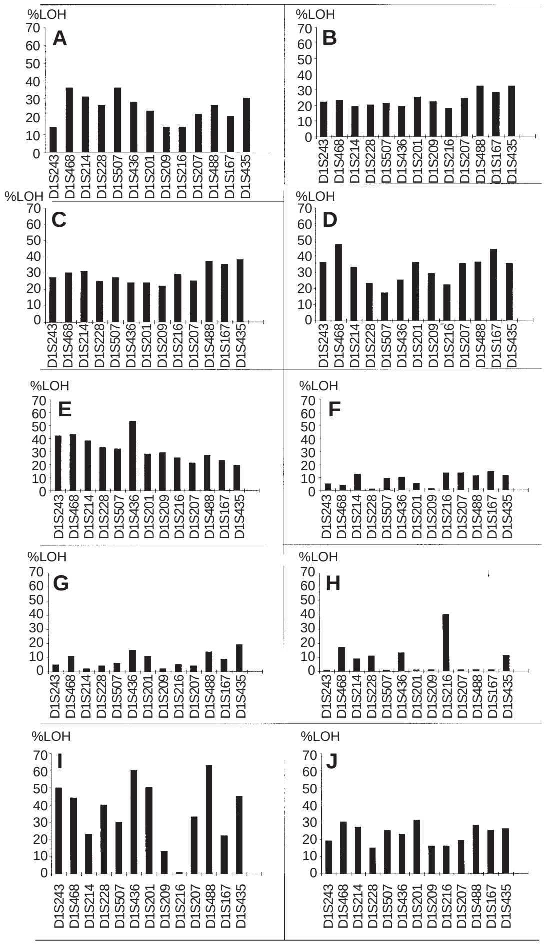

**Figure 3** Graphic illustrations of % LOH at 1p for individual markers in 10 different solid human cancer types: **A**, stomach (*n* = 38); **B**, colorectal (*n* = 10a) **C**, lung (*n* = 63); **D**, endometrial (*n* = 25); **E**, ovarian (*n* = 31) **F**, testis (*n* = 30); **G**, renal (*n* = 73); **H**, thyroid (*n* = 14); **I**, sarcoma (*n* = 14); and **J**, breast (*n* = 238)

SROs detected in the other three tumour types (Figure 4). A difference is also detected between the renal tumours and the other tumour types in the 1p32 region. In the 1p31 region the same SRO are detected, located between markers D1S435 and D1S167, in all four tumour types (Figure 4).

### *Association with clinical and pathological variables*

Chi-squared analysis comparing tumours with LOH at chromosome 1p with clinical and pathological variables showed a weak association between LOH at 1p and high S-phase fraction ($P$ = 0.021) in the breast tumours. There was also an association between LOH at 1p and poor tumour differentiation (grade III) in the colorectal cancer cases ($P$ = 0.047), but association was not found in other cancers (lung, stomach, endometrium, ovary, kidney or testis). There was no significant association between LOH at 1p and node status or tumour size in any cancer types (breast, lung, colorectum, stomach, endometrium, ovary, kidney or testis). There was no association between LOH at 1p in the breast tumours and parameters such as histological type, age at diagnosis, ploidy, or steroid receptor content. Furthermore, there was no association between LOH at 1p and Duke's grade in colorectal cancer cases (data not shown).

### *Survival analysis*

Breast cancer patients showed significant association between LOH at chromosome 1p and overall survival as tested with a log-rank test ($P < 0.001$). There was a significant separation of survival curves (Figure 5A). Median follow-up time is 5.0 years. When individual markers were analysed separately, with respect to association with breast cancer patients' overall survival, only one marker showed significance with a long-rank test ($P$ = 0.022) – the D1S435 marker, located at the chromosome region 1p31.1. Colorectal cancer patients showed a trend between LOH at 1p and survival ($P$ = 0.085) but the median follow-up time was only 2.1 years. Figure 5A shows the graphic representation of the survival statistics in the breast cancer patients and Figure 5B shows the survival statistics in colorectal cancer patients. Only 66 of the 146 breast cancer patients with LOH at 1p had metastasis in lymph nodes but 80 were lymph node-negative. Multivariate analysis was undertaken to evaluate the possible clinical relevance of LOH at 1p as a prognostic factor in breast cancer patients. The analysis showed that breast cancer patients with tumours with LOH at 1p had nearly a three-fold increase in relative mortality rate compared with patients with tumours without LOH at 1p. The RR (relative risk of dying in the multivariate analysis) was 2.7 (95% confidence interval: 1.5–4.9) and $P < 0.001$. The only other factor of prognostic value in the multivariate analysis was axillary nodal involvement with RR = 1.8 (95% CI: 1.1–3.0) and $P$ = 0.015 (Table 2A). A multivariate analysis in colorectal cancer patients showed that patients with LOH at 1p had a nearly twofold increase in relative mortality rate compared with patients with tumours without LOH at 1p. The 95% confidence interval of the multivariate analysis was 0.99–3.6 and $P$ = 0.055 (Table 2B).

### DISCUSSION

In this study LOH at chromosome 1p was detected with at least one marker in 54% of the tumours examined. The elevated frequency of LOH with certain markers suggests five regions of deletion indicating locations of putative TSGs. Known genes, located in these regions, that could possibly play a role in tumorigenesis are shown in Figure 2. The p*53* homologue p*73* on 1p36.33 (Kaghad et al, 1997) is one of these genes. A modifier gene of colon tumorigenesis (Mom1) in the mouse, which encodes secretory type II phospholipase $A_2$, affects the numbers of polyps developing as the consequence of a mutation in the *Apc* gene (Min mouse). A human homologue of the candidate for the *Mom*1 locus has been mapped to 1p35–p36.1 (Praml et al, 1995). Other genes or homologues to genes involved in cell growth and fidelity are: transcription factors, *E*2*F*2 on 1p36.11 (Lees et al, 1993), *PAX*7 on 1p36.1 (Vorobyov et al, 1997) and L-*myc* on 1p34.3 (Speleman et al, 1996); replication protein gene *RPA*2 on 1p35 (Ozawa et al, 1993); death receptor genes, *TR*2 (Kwon et al, 1997), *TNFR*2 (Kemper et al, 1991), *DR*3 (Bodmer et al, 1997) and *DR*5 (Wu et al, 1997), all on 1p36; other receptor genes, *PTAFR* on 1p34.3–p35 (Chase et al, 1996), *TGF*β*R*3 on 1p32–p33 (Johnson et al, 1995) and *IL*12*R*β2 on 1p31.2 (Yamamoto et al, 1997); prostaglandin receptors, *PTGER*3 on 1p31.2 and *PTGFR* on 1p31.1 (Duncan et al, 1995); tyrosine kinases, *ECK* on 1p36.1 (Sulman et al, 1997), *LCK* on 1p35 (Volpi et al, 1994) and *JAK*1 on 1p31.3–p32.3 (Modi et al, 1995); dual specific kinase *ERK* (Saito et al, 1995) on 1p36.1; repair protein genes, *RAD*54 on 1p32 (Rasio et al, 1997); and *GADD*45 on 1p31.1–p31.2 (Hey et al, 1996); cell cycle control proteins,

© Cancer Research Campaign 1999

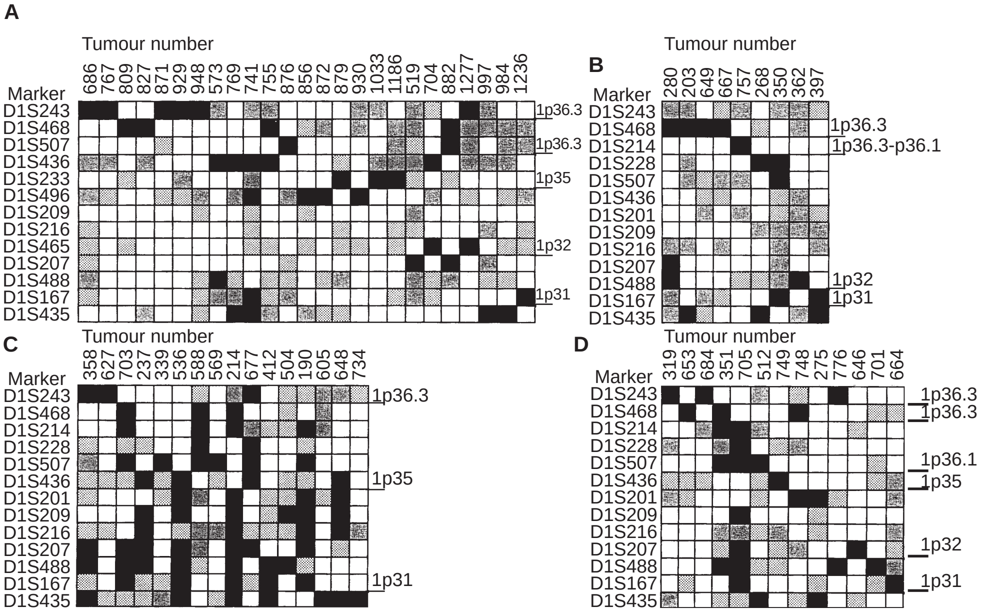

Figure 4 Patterns of LOH in selected samples of (**A**) breast, (**B**) renal, (**C**) lung and (**D**) colorectal tumours. Black, LOH; white, ROH (retention of heterozygosity); gridline, homozygous; grey, not informative. These are selected tumours with partial and interstitial deletions on chromosome 1p. The complex pattern of LOH could be consistent with the smallest regions of overlap (SROs), in colorectal and lung tumours, in regions 1p36.3 (6.4 cM), 1p36.1 (4.5 cM), 1p35–p34.3 (23.1 cM), 1p32 (0.5 cM) and 1p31 (3.4 cM). In breast tumours microdeletions are consistent with SRO at the same regions. In renal tumours the deletion pattern is different to other tumour types in the distal as well as the more proximal regions. The SRO at the 1p31 region is located between the same markers in renal tumours as in the other tumour types but other SROs map differently; the SROs in the renal tumours are located in the 1p36.3 region between markers D1S468 and D1S214 (10.4 cM), in the 1p36.3–1p36.1 region between markers D1S214 and D1S228 (16.4 cM), and in the 1p32–p31.3 region between markers D1S488 and D1S167 (7.4 cM)

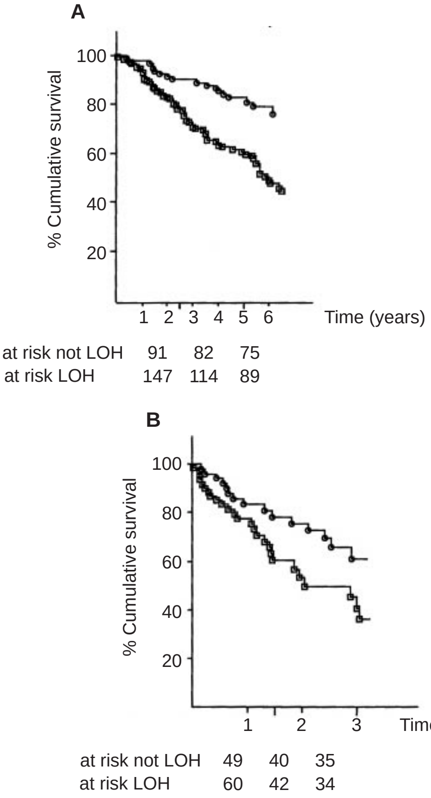

Figure 5 Cumulative percent surviving at different time intervals for (**A**) breast cancer patients and (**B**) colorectal cancer patients. Time intervals are given in years. The median follow-up time is (**A**) 5.0 years and (**B**) 2.1 years. The upper line shows patients without LOH on 1p but the lower line shows patients with LOH on 1p. The number of patients at risk at (**A**) time 0, 2, 5 years, and (**B**) 0, 1.5 and 3 years, are shown for both categories

*CDC2L1* (Lahti et al, 1994) on 1p36.2, *RIZ* on 1p36.1 (Buyse et al, 1996) and *p18* (*INK4C*) on 1p32 (Lapointe et al, 1996).

A difference in LOH was detected between different tumour types. Some tumour types, testis and renal cancer, show low frequency of LOH while other tumour types have a high frequency of LOH, the highest in sarcomas and endometrial cancers. A subset of markers show elevated frequency of LOH in a number of different tumour types, suggesting that loss of a given TSG might be involved in pathogenesis of tumour growth in different tissues. Otherwise, a distinct pattern of LOH was detected in individual tumour types, reflecting multiple TSGs, that could be differentially inactivated in tumorigenesis. Furthermore, the study shows that LOH at 1p can be detected in tumours of the mouth, oesophagus, liver, pancreas, prostate, brain and adrenal gland, as well as melanoma and lymphoma.

There was a weak significant association between LOH at 1p and high S-phase fraction in breast tumours and between LOH and poor tumour differentiation in colorectal tumours. In breast cancer patients, there seems to be no association between LOH at 1p and age of onset, tumour ploidy or steroid receptor content. Furthermore, no association was detected between LOH at 1p and tumour size or nodal metastasis in tumours of the breast, stomach, colorectum, lung, endometrium, ovary, kidney or testis. LOH at 1p does not appear to be associated with the nodal metastasis step in carcinogenesis. LOH at 1p is an independent prognostic factor for the breast cancer patients and is in line with our earlier finding with less patient material and a shorter follow-up time (Ragnarsson et al, 1996). Furthermore, LOH at 1p seems to be a prognostic factor in colorectal cancer also, though in this study the association is not significant probably due to the short follow-up time. In a different study it was concluded that allelic loss in the

1p36 and 1p32 regions of chromosome 1 appears to be an independent predictor of poor prognosis in patients with adenocarcinoma of the colon (Ogunbiyi et al, 1997).

### ACKNOWLEDGEMENTS

The authors wish to thank Professor Dr J Hallgrimsson and his staff at the Department of Pathology, University Hospital of Iceland for providing pathological information. We also wish to thank the Icelandic Cancer Society, University Science Fund and the Icelandic Research Council for financial support.

### REFERENCES

Arlt MF, Herzog TJ, Mutch DG, Gersell DJ, Liu H and Goodfellow PJ (1996) Frequent deletions of chromosome 1p sequences of aggressive histologic subtype of endometrial cancer. *Hum Mol Genet* **5**: 1017–1021

Bardi G, Pandis N, Fenger C, Kronborg O, Bomme L and Heim S (1993) Deletion of 1p36 as primary chromosomal aberration in intestinal tumorigenesis. *Cancer Res* **53**: 1895–1898

Bello MJ, de Campos JM, Kusak ME, Vaquero J, Sarasa JL, Pestana A and Rey JA (1994) Allelic loss at 1p is associated with tumor progression of meningiomas. *Genes Chromosom Cancer* **9**: 296–298

Bieche I, Champème MH and Lidereau R (1994) A tumor suppressor gene on chromosome 1p32-pter controls the amplification of MYC family genes in breast cancer. *Cancer Res* **54**: 4274–4276

Bodmer JL, Burns K, Sneider P, Hofmann K, Steiner V, Thome M, Bornand T, Hahne M, Schroter M, Becker K, Wilson A, French LE, Browning JL, MacDonald HR and Tschopp J (1997) Tramp, a novel apotosis-mediating receptor with sequence homology to tumor necrosis factor receptor 1 and Fas(Apo-1/CD95). *Immunity* **6**: 79–88

Buyse IM, Takahashi EI and Huang S (1996) Physical mapping of retinoblastoma interacting zinc finger gene RIZ to D1S228 on chromosome 1p36. *Genomics* **15**: 119–121

Caron H, Peter M, van Sluis P, Speleman F, de Kraker J, Laureys G, Michon J, Brugieres L, Voute PA, Westerveld A, Slater R, Delattre O and Versteeg R (1995) Evidence for two tumour suppressor loci on chromosomal bands 1p35–36 involved in neuroblastoma: one probably imprinted, another associated with N-myc amplification. *Hum Mol Genet* **4**: 535–539

Caron H, van Sluis P, de Kraker J, Bokkerink J, Egeler M, Laureys G, Slater R, Westerveld A, Voute PA and Versteeg R (1996) Allelic loss of chromosome 1p as a predictor of unfavourable outcome in patients with neuroblastoma. *N Engl J Med* **334**: 225–230

Cavenee WK and White RL (1995) The genetic basis of cancer. An accumulation of genetic defects can apparently cause normal cells to become cancerous and cancerous cells to become increasingly dangerous. *Sci Am* (March) 72–79

Chase PB, Yang JM, Thompson FH, Halonen M and Regan JW (1996) Regional mapping of the platelet-activating factor receptor gene (PTAFR) to 1p35–p34.3 by fluorescence in situ hybridisation. *Cytogenet Cell Genet* **72**: 205–207

Chen LC, Kurisu W, Ljung BM, Goldman ES, Moore D II and Smith HS (1992) Heterogeneity for allelic loss in human breast cancer. *J Natl Cancer Inst* **84**: 506–510

Cox DR (1972) Regression models and life-tables. *J R Stat Soc (B)* **34**: 187–220

Di Vinci A, Infusini E, Peveri C, Risio C, Rossini FP and Giaretti W (1996) Deletions at chromosome 1p by fluorescence in situ hybridisation are an early event in human colorectal tumorigenesis. *Gastroenterology* **111**: 102–107

Dracopoli NC, Harnett P, Bale SJ, Stanger BZ, Tucker MA, Housman DE and Kefford RF (1989) Loss of alleles from the distal short arm of chromosome 1 occurs late in melanoma tumor progression. *Proc Natl Acad Sci USA* **86**: 4614–4618

Duncan AMV, Anderson LL, Funk CD, Abramovitz M and Adam M (1995) Chromosomal localisation of the prostanoid receptor gene family. *Genomics* **25**: 740–742

Ezaki T, Yanagisawa A, Otha K, Aiso S, Watanabe M, Hibi T, Kato Y, Nakajima T, Ariyama T, Inazawa J, Nakamura Y and Horii A (1996) Deletions mapping on chromosome 1p in well-differentiated gastric cancer. *Br J Cancer* **73**: 424–428

Haber D and Harlow E (1997) Tumor-suppressor genes: evolving definitions in the genomic age. *Nat Genet* **16**: 320–322

Hey Y, Hoggard N, Brintnell B, James L, Jones D, Mitchell E, Weissenbach J and Varley JM (1996) Identification and cloning in yeast artificial chromosomes of a region of elevated loss of heterozygosity on chromosome 1p31.1 in human breast cancer. *Cytogenet Cell Genet* **72**: 148

Johnson DW, Qumsiyeh M and Benkhalifa M (1995) Assignment of human transforming growth factor-beta type I and type III receptor genes (TGFRBR1 and TGFBR3) to 9q33-q34 and 1p32–p33, respectively. *Genomics* **28**: 356–357

Kaghad M, Bonnet H, Yang A, Creancier L, Biscan JC, Valent A, Minty A, Chalon P, Lelias JM, Dumont X, Ferrara P, McKeon F and Caput D (1997) Monoallelically expressed gene related to p53 at 1p36, a region frequently deleted in neuroblastoma and other human cancers. *Cell* **90**: 809–819

Kaplan EL and Meier P (1958) Nonparametric estimation from incomplete observations. *J Am Stat Assoc* **53**: 457–481

Kemper O, Derre J, Cherif D, Engelmann H, Wallach D and Berger R (1991) The gene for the type II (p75) tumor necrosis factor receptor (TNF-RII) is localized on band 1p36.2–p36.3. *Hum Genet* **87**: 623–624

Kuroki T, Fujiwara Y, Tsuchiya E, Nakamori S, Imaoka S, Kanematsu T and Nakamura Y (1995) Accumulation of genetic changes during development and progression of hepatocellular carcinoma: loss of heterozygosity of chromosome arm 1p occurs at an early stage of hepatocarcinogenesis. *Genes Chromosom Cancer* **13**: 163–167

Kwon BS, Tan KB, Ni J, Lee KO, Kim KK, Kim YJ, Wang S, Gentz R, Yu GL, Harrop J, Lyn SD, Silverman C, Porter TG, Truneh A and Young PR (1997) A newly identified member of the tumor necrosis factor receptor superfamily with a wide tissue distribution and involvement in lymphocyte activation. *J Biol Chem* **272**: 14272–14276

Lahti JM, Valentine M, Xiang J, Jones B, Amann J, Grenet J, Richmond G, Look AT and Kidd VJ (1994) Alteration in the PITSLRE protein kinase gene complex on chromosome 1p36 in childhood neuroblastoma. *Nat Genet* **7**: 370–375

Lapointe J, Lachance Y and Labrie C (1996) A p18 mutant defective in cdk6 binding in human breast cancer cells. *Cancer Res* **56**: 4586–4589

Lees JA, Saito M, Vidal M, Valentine M, Look T, Harlow E, Dyson N and Helin K (1993) The retinoblastoma protein binds to a family of E2F transcriptional factors. *Mol Cell Biol* **13**: 7813–7825

Mantel N (1960) Evaluation of survival data and two new rank order statistics arising in its consideration. *Cancer Chemother Rep* **50**: 163–170

Mathew CGP, Smith BA, Thorpe K, Wong Z, Royle NJ, Jeffreys AJ and Ponder BAJ (1987) Deletion of genes on chromosome 1 in endocrine neoplasia. *Nature* **328**: 524–526

Mathew S, Murty VV, Bosl GJ and Chaganti RS (1994) Loss of heterozygosity identifies multiple sites of allelic deletions on chromosome 1 in human male germ cell tumors. *Cancer Res* **54**: 6265–6269

Miller SA, Dykes DD and Polesky HF (1988) A simple salting out procedure for extracting DNA from human nucleated cells. *Nucl Acids Res* **16**: 1215

Modi WS, Farrar WL and Howard OMZ (1995) Confirmed assignment of a novel human tyrosine kinase gene (JAK1A) to 1p32.3–31.3 by non isotopic in situ hybridization. *Cytogenet Cell Genet* **69**: 232–234

Munn KE, Walker RA and Varley JM (1995) Frequent alterations of chromosome 1 in ductal carcinoma in situ of the breast. *Oncogene* **10**: 1653–1657

Ogunbiyi OA, Goodfellow PJ, Gagliardi G, Swanson PE, Birnbaum EH, Fleshman JW, Kodner IJ and Moley JF (1997) Prognostic value of chromosome 1p allelic loss in colon cancer. *Gastroenterology* **113**: 761–766

Ozawa K, Dean FB, Chen M, Lee SH, Shiratori A, Murakami Y, Sakura T, Hurwitz J and Eki T (1993) Mapping of the 70 kDa, 34 kDa, and a 11 kDa subunit of the human multimeric single-stranded DNA binding protein /hSSB/RPA) to chromosome bands 17p13, 1p35–p36.1, and 7p21–p22. *Cell Struct Funct* **18**: 221–230

Praml C, Savelyeva L, Le Paslier D, Siracusa LD, Buchberg AM, Schwab M and Amler LC (1995) Human homologue of a candidate for the MOM1 locus, the secretory type II phospholipase $A_2$ (PLA2S-II), maps to 1p35–36.1/D1S199. *Cancer Res* **55**: 5504–5506

Ragnarsson G, Sigurdsson A, Eiriksdottir G, Barkardottir RB, Jonasson JG and Ingvarsson S (1996) Loss of heterozygosity at chromosomes 1p in human breast cancer – association with high S-phase, reduced patients survival, and deletions at other chromosome regions. *Int J Oncol* **9**: 731–736

Rasio D, Murakumo Y, Robbins D, Roth T, Silver A, Negrini M, Schmidt C, Burczak J, Fishel R and Croce CM (1997) Characterisation of the human homologue of RAD54 — a gene located on chromosome 1p32 at a region of high loss of heterozygosity in breast tumors. *Cancer Res* **57**: 2378–2383

Saito T, Seki N, Matsuda Y, Kitahara M, Murata M, Kanda N, Nomura N, Yamamoto T and Hori TA (1995) Identification of the human ERK gene as a putative receptor tyrosine kinase and its chromosomal localisation to 1p36.1: a comparative mapping of human, mouse, and rat chromosomes. *Genomic* **26**: 382–384

© Cancer Research Campaign 1999

Smith SA, Easton DF, Evans DG and Ponder BA (1992) Allele losses in the region 17q12–21 in familial breast and ovarian cancer involve the wild-type chromosome. *Nature Genet* **2**: 128–131

Speleman F, Van Camp G and Van Roy N (1996) Reassignment of MYCL1 to human chromosome 1p34.3 by fluorescence in situ hybridization. *Cytogenet Cell Genet* **72**: 189–190

Steenman M, Redeker B, de Meulemeester M, Wiesmeijer K, Voute PA, Westerveld A, Slater R and Mannens M (1997) Comparative genomic hybridization analysis of Wilms tumor. *Cytogenet Cell Genet* **77**: 296–303

Stock C, Ambros IM, Lion T, Haas OA, Zoubek A, Gadner H and Ambros PF (1994) Detection of numerical and stuctural chromosome abnormalities in paediatric germ cell tumors by means of interphase cytogenetics. *Genes Chromosomes Cancer* **11**: 40–50

Sulman EP, Tang XX, Allen C, Biegel JA, Pleasure DE, Brodeur GM and Ikegaki N (1997) ECK, a human EPH-related gene maps to 1p36.1, a common region of alteration in human cancer. *Genomics* **40**: 371–374

Taguchi T, Jhanwar SC, Siegfried JM, Keller SM and Testa JR (1993) Recurrent deletions of specific chromosomal sites in 1p, 3p, 6q and 9p in human malignant mesothelioma. *Cancer Res* **53**: 4349–4355

Tanaka K, Yanoshita R, Konishi M, Oshimura M, Maeda Y, Mori T and Miyaki M (1993) Suppression of tumorigenicity in human colon carcinoma cell by introduction of normal chromosome 1p36 region. *Oncogene* **8**: 2253–2258

Vargas MP, Zhuang Z, Wang C, Vortmeyer A, Linehan WM and Merino MJ (1997) Loss of heterozygosity on the short arm of chromosomes 1 and 3 in sporadic pheochromocytoma and extra-adrenal paraganglioma. *Hum Pathol* **28**: 411–415

Volpi EV, Romani M and Siniscalco M (1994) Subregional mapping of the human lymphocyte-specific protein tyrosine kinase gene (LCK) to 1p35–p34.3 and its position relative to the 1p marker D1S57. *Cytogenet Cell Genet* **67**: 187–189

Vorobyov E, Mertsalov I, Dockhorn-Dwoeniczak B, Dwoeniczak B and Horst J (1997) The genomic organization and the full coding region of the human PAX7 gene. *Genomics* **45**: 168–174

White PS, Maris JM, Beltinger C, Sulman E, Marshall HN, Fujimori M, Kaufman BA, Biegel JA, Allen C, Hilliard C, Valentine MB, Look AT, Enomoto H, Sakiyama S and Brodeur GM (1995) A region of consistent deletion in neuroblastoma maps within human chromosome 1p36.2–36.3. *Proc Natl Acad Sci USA* **92**: 5520–5524

Wu GS, Burns TF, McDonald ER, Jiang W, Meng R, Krantz ID, Kao G, Gan DD, Zhou JY, Muchel R, Hamilton SR, Spinner NB, Markowitz S, Wu G and el-Deiry WS (1997) Killer/DR5 is a DNA damage-inducible p53-regulated death receptor gene. *Nat Genet* **17**: 141–143

Yamamoto K, Kobayashi H, Miura O, Hirosawa S and Miyasaka N (1997) Assignment of IL12RB1 and IL12RB2, interleukin-12 receptor beta1 and beta2 chains, to human chromosome 19 band p13.1 and chromosome 1 band p31.2, respectively, by in situ hybridization. *Cytogenet Cell Genet* **77**: 257–258

Yeh SH, Chen PJ, Chen HL, Lai MY, Wang CC and Chen DS (1994) Frequent genetic alterations at the distal region of chromosome 1p in human hepatocellular carcinomas. *Cancer Res* **54**: 4188–4192

*© Cancer Research Campaign 1999*

## References

[PDF_00878] Arlt M. F., Herzog T. J., Mutch D. G., Gersell D. J., Liu H., Goodfellow P. J. (1996). Frequent deletion of chromosome 1p sequences in an aggressive histologic subtype of endometrial cancer.. Hum Mol Genet.

[PDF_00881] Bardi G., Pandis N., Fenger C., Kronborg O., Bomme L., Heim S. (1993). Deletion of 1p36 as a primary chromosomal aberration in intestinal tumorigenesis.. Cancer Res.

[PDF_00884] Bello M. J., de Campos J. M., Kusak M. E., Vaquero J., Sarasa J. L., Pestaña A., Rey J. A. (1994). Allelic loss at 1p is associated with tumor progression of meningiomas.. Genes Chromosomes Cancer.

[PDF_00887] Bièche I., Champème M. H., Lidereau R. (1994). A tumor suppressor gene on chromosome 1p32-pter controls the amplification of MYC family genes in breast cancer.. Cancer Res.

[PDF_00890] Bodmer J. L., Burns K., Schneider P., Hofmann K., Steiner V., Thome M., Bornand T., Hahne M., Schröter M., Becker K. (1997). TRAMP, a novel apoptosis-mediating receptor with sequence homology to tumor necrosis factor receptor 1 and Fas(Apo-1/CD95).. Immunity.

[PDF_00895] Buyse I. M., Takahashi E. I., Huang S. (1996). Physical mapping of the retinoblastoma interacting zinc finger gene RIZ to D1S228 on chromosome 1p36.. Genomics.

[PDF_00898] Caron H., Peter M., van Sluis P., Speleman F., de Kraker J., Laureys G., Michon J., Brugières L., Voûte P. A., Westerveld A. (1995). Evidence for two tumour suppressor loci on chromosomal bands 1p35-36 involved in neuroblastoma: one probably imprinted, another associated with N-myc amplification.. Hum Mol Genet.

[PDF_00903] Caron H., van Sluis P., de Kraker J., Bökkerink J., Egeler M., Laureys G., Slater R., Westerveld A., Voûte P. A., Versteeg R. (1996). Allelic loss of chromosome 1p as a predictor of unfavorable outcome in patients with neuroblastoma.. N Engl J Med.

[PDF_00911] Chase P. B., Yang J. M., Thompson F. H., Halonen M., Regan J. W. (1996). Regional mapping of the human platelet-activating factor receptor gene (PTAFR) to 1p35-->p34.3 by fluorescence in situ hybridization.. Cytogenet Cell Genet.

[PDF_00914] Chen L. C., Kurisu W., Ljung B. M., Goldman E. S., Moore D., Smith H. S. (1992). Heterogeneity for allelic loss in human breast cancer.. J Natl Cancer Inst.

[PDF_00918] Di Vinci A., Infusini E., Peveri C., Risio M., Rossini F. P., Giaretti W. (1996). Deletions at chromosome 1p by fluorescence in situ hybridization are an early event in human colorectal tumorigenesis.. Gastroenterology.

[PDF_00921] Dracopoli N. C., Harnett P., Bale S. J., Stanger B. Z., Tucker M. A., Housman D. E., Kefford R. F. (1989). Loss of alleles from the distal short arm of chromosome 1 occurs late in melanoma tumor progression.. Proc Natl Acad Sci U S A.

[PDF_00925] Duncan A. M., Anderson L. L., Funk C. D., Abramovitz M., Adam M. (1995). Chromosomal localization of the human prostanoid receptor gene family.. Genomics.

[PDF_00928] Ezaki T., Yanagisawa A., Ohta K., Aiso S., Watanabe M., Hibi T., Kato Y., Nakajima T., Ariyama T., Inazawa J. (1996). Deletion mapping on chromosome 1p in well-differentiated gastric cancer.. Br J Cancer.

[PDF_00931] Haber D., Harlow E. (1997). Tumour-suppressor genes: evolving definitions in the genomic age.. Nat Genet.

[PDF_00937] Johnson D. W., Qumsiyeh M., Benkhalifa M., Marchuk D. A. (1995). Assignment of human transforming growth factor-beta type I and type III receptor genes (TGFBR1 and TGFBR3) to 9q33-q34 and 1p32-p33, respectively.. Genomics.

[PDF_00940] Kaghad M., Bonnet H., Yang A., Creancier L., Biscan J. C., Valent A., Minty A., Chalon P., Lelias J. M., Dumont X. (1997). Monoallelically expressed gene related to p53 at 1p36, a region frequently deleted in neuroblastoma and other human cancers.. Cell.

[PDF_00946] Kemper O., Derré J., Cherif D., Engelmann H., Wallach D., Berger R. (1991). The gene for the type II (p75) tumor necrosis factor receptor (TNF-RII) is localized on band 1p36.2-p36.3.. Hum Genet.

[PDF_00949] Kuroki T., Fujiwara Y., Tsuchiya E., Nakamori S., Imaoka S., Kanematsu T., Nakamura Y. (1995). Accumulation of genetic changes during development and progression of hepatocellular carcinoma: loss of heterozygosity of chromosome arm 1p occurs at an early stage of hepatocarcinogenesis.. Genes Chromosomes Cancer.

[PDF_00954] Kwon B. S., Tan K. B., Ni J., Oh K. O., Lee Z. H., Kim K. K., Kim Y. J., Wang S., Gentz R., Yu G. L. (1997). A newly identified member of the tumor necrosis factor receptor superfamily with a wide tissue distribution and involvement in lymphocyte activation.. J Biol Chem.

[PDF_00959] Lahti J. M., Valentine M., Xiang J., Jones B., Amann J., Grenet J., Richmond G., Look A. T., Kidd V. J. (1994). Alterations in the PITSLRE protein kinase gene complex on chromosome 1p36 in childhood neuroblastoma.. Nat Genet.

[PDF_00962] Lapointe J., Lachance Y., Labrie Y., Labrie C. (1996). A p18 mutant defective in CDK6 binding in human breast cancer cells.. Cancer Res.

[PDF_00964] Lees J. A., Saito M., Vidal M., Valentine M., Look T., Harlow E., Dyson N., Helin K. (1993). The retinoblastoma protein binds to a family of E2F transcription factors.. Mol Cell Biol.

[PDF_00967] Mantel N. (1966). Evaluation of survival data and two new rank order statistics arising in its consideration.. Cancer Chemother Rep.

[PDF_00969] Mathew C. G., Smith B. A., Thorpe K., Wong Z., Royle N. J., Jeffreys A. J., Ponder B. A. (1987). Deletion of genes on chromosome 1 in endocrine neoplasia.. Nature.

[PDF_00972] Mathew S., Murty V. V., Bosl G. J., Chaganti R. S. (1994). Loss of heterozygosity identifies multiple sites of allelic deletions on chromosome 1 in human male germ cell tumors.. Cancer Res.

[PDF_00975] Miller S. A., Dykes D. D., Polesky H. F. (1988). A simple salting out procedure for extracting DNA from human nucleated cells.. Nucleic Acids Res.

[PDF_00977] Modi W. S., Farrar W. L., Howard O. M. (1995). Confirmed assignment of a novel human tyrosine kinase gene (JAK1A) to 1p32.3-->p31.3 by nonisotopic in situ hybridization.. Cytogenet Cell Genet.

[PDF_00980] Munn K. E., Walker R. A., Varley J. M. (1995). Frequent alterations of chromosome 1 in ductal carcinoma in situ of the breast.. Oncogene.

[PDF_00982] Ogunbiyi O. A., Goodfellow P. J., Gagliardi G., Swanson P. E., Birnbaum E. H., Fleshman J. W., Kodner I. J., Moley J. F. (1997). Prognostic value of chromosome 1p allelic loss in colon cancer.. Gastroenterology.

[PDF_00985] Ozawa K., Dean F. B., Chen M., Lee S. H., Shiratori A., Murakami Y., Sakakura T., Hurwitz J., Eki T. (1993). Mapping of the 70 kDa, 34 kDa, and 11 kDa subunit genes of the human multimeric single-stranded DNA binding protein (hSSB/RPA) to chromosome bands 17p13, 1p35-p36.1, and 7p21-p22.. Cell Struct Funct.

[PDF_00990] Praml C., Savelyeva L., Le Paslier D., Siracusa L. D., Buchberg A. M., Schwab M., Amler L. C. (1995). Human homologue of a candidate for the Mom1 locus, the secretory type II phospholipase A2 (PLA2S-II), maps to 1p35-36.1/D1S199.. Cancer Res.

[PDF_00999] Rasio D., Murakumo Y., Robbins D., Roth T., Silver A., Negrini M., Schmidt C., Burczak J., Fishel R., Croce C. M. (1997). Characterization of the human homologue of RAD54: a gene located on chromosome 1p32 at a region of high loss of heterozygosity in breast tumors.. Cancer Res.

[PDF_01003] Saito T., Seki N., Matsuda Y., Kitahara M., Murata M., Kanda N., Nomura N., Yamamoto T., Hori T. A. (1995). Identification of the human ERK gene as a putative receptor tyrosine kinase and its chromosomal localization to 1p36.1: a comparative mapping of human, mouse, and rat chromosomes.. Genomics.

[PDF_01012] Smith S. A., Easton D. F., Evans D. G., Ponder B. A. (1992). Allele losses in the region 17q12-21 in familial breast and ovarian cancer involve the wild-type chromosome.. Nat Genet.

[PDF_01015] Speleman F., Van Camp G., Van Roy N. (1996). Reassignment of MYCL1 to human chromosome 1p34.3 by fluorescence in situ hybridization.. Cytogenet Cell Genet.

[PDF_01018] Steenman M., Redeker B., de Meulemeester M., Wiesmeijer K., Voûte P. A., Westerveld A., Slater R., Mannens M. (1997). Comparative genomic hybridization analysis of Wilms tumors.. Cytogenet Cell Genet.

[PDF_01021] Stock C., Ambros I. M., Lion T., Haas O. A., Zoubek A., Gadner H., Ambros P. F. (1994). Detection of numerical and structural chromosome abnormalities in pediatric germ cell tumors by means of interphase cytogenetics.. Genes Chromosomes Cancer.

[PDF_01025] Sulman E. P., Tang X. X., Allen C., Biegel J. A., Pleasure D. E., Brodeur G. M., Ikegaki N. (1997). ECK, a human EPH-related gene, maps to 1p36.1, a common region of alteration in human cancers.. Genomics.

[PDF_01028] Taguchi T., Jhanwar S. C., Siegfried J. M., Keller S. M., Testa J. R. (1993). Recurrent deletions of specific chromosomal sites in 1p, 3p, 6q, and 9p in human malignant mesothelioma.. Cancer Res.

[PDF_01031] Tanaka K., Yanoshita R., Konishi M., Oshimura M., Maeda Y., Mori T., Miyaki M. (1993). Suppression of tumourigenicity in human colon carcinoma cells by introduction of normal chromosome 1p36 region.. Oncogene.

[PDF_01034] Vargas M. P., Zhuang Z., Wang C., Vortmeyer A., Linehan W. M., Merino M. J. (1997). Loss of heterozygosity on the short arm of chromosomes 1 and 3 in sporadic pheochromocytoma and extra-adrenal paraganglioma.. Hum Pathol.

[PDF_01038] Volpi E. V., Romani M., Siniscalco M. (1994). Subregional mapping of the human lymphocyte-specific protein tyrosine kinase gene (LCK) to 1p35-->p34.3 and its position relative to the 1p marker D1S57.. Cytogenet Cell Genet.

[PDF_01041] Vorobyov E., Mertsalov I., Dockhorn-Dworniczak B., Dworniczak B., Horst J. (1997). The genomic organization and the full coding region of the human PAX7 gene.. Genomics.

[PDF_01044] White P. S., Maris J. M., Beltinger C., Sulman E., Marshall H. N., Fujimori M., Kaufman B. A., Biegel J. A., Allen C., Hilliard C. (1995). A region of consistent deletion in neuroblastoma maps within human chromosome 1p36.2-36.3.. Proc Natl Acad Sci U S A.

[PDF_01051] Wu G. S., Burns T. F., McDonald E. R., Jiang W., Meng R., Krantz I. D., Kao G., Gan D. D., Zhou J. Y., Muschel R. (1997). KILLER/DR5 is a DNA damage-inducible p53-regulated death receptor gene.. Nat Genet.

[PDF_01053] Yamamoto K., Kobayashi H., Miura O., Hirosawa S., Miyasaka N. (1997). Assignment of IL12RB1 and IL12RB2, interleukin-12 receptor beta 1 and beta 2 chains, to human chromosome 19 band p13.1 and chromosome 1 band p31.2, respectively, by in situ hybridization.. Cytogenet Cell Genet.

[PDF_01057] Yeh S. H., Chen P. J., Chen H. L., Lai M. Y., Wang C. C., Chen D. S. (1994). Frequent genetic alterations at the distal region of chromosome 1p in human hepatocellular carcinomas.. Cancer Res.

